# Identification of novel alleles of the rice blast resistance gene *Pi54*

**DOI:** 10.1038/srep15678

**Published:** 2015-10-26

**Authors:** Kumar Vasudevan, Wilhelm Gruissem, Navreet K. Bhullar

**Affiliations:** 1 Plant Biotechnology, Department of Biology, ETH Zurich (Swiss Federal Institute of Technology), Zurich, Switzerland

## Abstract

Rice blast is one of the most devastating rice diseases and continuous resistance breeding is required to control the disease. The rice blast resistance gene *Pi54* initially identified in an Indian cultivar confers broad-spectrum resistance in India. We explored the allelic diversity of the *Pi54* gene among 885 Indian rice genotypes that were found resistant in our screening against field mixture of naturally existing *M. oryzae* strains as well as against five unique strains. These genotypes are also annotated as rice blast resistant in the International Rice Genebank database. Sequence-based allele mining was used to amplify and clone the *Pi54* allelic variants. Nine new alleles of *Pi54* were identified based on the nucleotide sequence comparison to the *Pi54* reference sequence as well as to already known *Pi54* alleles. DNA sequence analysis of the newly identified *Pi54* alleles revealed several single polymorphic sites, three double deletions and an eight base pair deletion. A SNP-rich region was found between a tyrosine kinase phosphorylation site and the nucleotide binding site (NBS) domain. Together, the newly identified *Pi54* alleles expand the allelic series and are candidates for rice blast resistance breeding programs.

Rice is a staple food for more than half of the world’s population and therefore important for global food security. Rice blast, which is caused by the fungus *Magnaporthe oryzae*, is one of the most devastating rice diseases worldwide. The rice blast infection can damage almost all parts of the plant at various growth stages^1^,^2^. Frequent epidemics and outbreaks of rice blast were reported in different major rice growing countries, with yield losses ranging from 20 to 100%^1^,^3^. Considering the severity of the disease and its significant contribution to the rice yield gap, extensive research in different laboratories is addressing various aspects essential for management of the disease, including genomics, infection mechanism, host-pathogen interactions and resistance breeding.

Deployment of host plant resistance is the most effective approach for the control of rice blast. To date nearly 100 blast resistance (*R*) genes and over 350 quantitative trait loci (QTLs) have been identified^1^, of which 21 have been cloned and characterized in detail^4^,^5^,^6^,^7^,^8^. Molecular marker information is facilitating marker-assisted selection (MAS) for incorporating these blast resistance genes into rice breeding programs. Examples of successful deployment of blast *R* genes include the introgression of *Piz5* and *Pi54* into an elite basmati rice restorer line ‘PRR78’^9^ and the development of pyramided lines with *Pib* and *Pish*^10^ as well as *Pi1*, *Piz-5* and *Pita* combinations^11^ in the cultivar CO39 background. Despite the availability of several cloned *R* genes, rice blast remains problematic because resistance is often lost in new varieties after a few years of intensive agricultural use. The rapidly evolving blast fungus can overcome the resistance conferred by major blast resistance genes. Because of the importance of the disease, *M. oryzae* was the first phytopathogenic fungus whose genome was sequenced. The *M. oryzae* genome contains retrotransposons and abundant repetitive elements in regions carrying the avirulence (*Avr)* genes^12^. To better understand the genetic variation among *M. oryzae* strains, genomes of three additional *M. oryzae* strains were re-sequenced^13^,^14^. This together with recent studies on blast *Avr* genes revealed high levels of non-synonymous variations as well as frequent loss and gain of *Avr* genes, which explains the rapid evolving nature of the blast fungus^13^,^15^.

Pertaining to the genetic variability and pathogenicity of the rice blast fungus, genes involved in host plant resistance also co-evolved with high levels of allelic and copy number variations^16^. Many of the major blast resistance genes are located in complex gene clusters on chromosomal loci harbouring multiple other blast resistance genes. Several of these genes also exist in multiple copy numbers, serving as evidence for the natural selection-driven *R* gene diversification^17^,^18^. Moreover, minor sequence variations among blast *R* genes, such as the presence of a few single nucleotide polymorphisms (SNPs), lead to major differences in their function or resistance spectrum. For instance, only few amino acid changes among the predicted proteins of blast resistance genes *Pi2*, *Pi9* and *Piz-t* determine their resistance specificities^19^, with each of them conferring broad-spectrum resistance against different rice blast isolates^20^. In addition to the resistance spectrum difference among individual blast resistance genes, different allelic forms of major *R* genes also often have varied specificities. For example, a single amino acid change differentiates the resistant and susceptible alleles of rice blast resistance genes *Pita* and *Pid2*^21^,^22^. Likewise, alleles of the powdery mildew resistance *Pm3* gene in wheat exhibit different resistance patterns against a range of powdery mildew isolates^23^,^24^,^25^. Combining such alleles of a major *R* gene in a single genetic background or through multiline approach is a possible strategy to improve resistance in the field^26^,^27^. The presence of multiple allelic forms might reduce the selection pressure on the pathogen, thereby increasing the durability of resistance^5^. In addition, intragenic pyramiding that combines the specificities of different alleles is an alternative to broaden the recognition spectrum as compared to the parental alleles^25^. This has been successfully demonstrated in the case of *Pm3* where the specificities of *Pm3d* and *Pm3e* were combined into a single allele. Moreover, a detailed molecular analysis of such allelic *R* gene variants facilitated understanding of host-pathogen interactions and evolution of disease resistance genes. Therefore, exploring naturally available genetic diversity for new alleles of resistance genes is an efficient approach for broadening the resistance sources against rice blast.

Seed banks are rich sources of such natural genetic diversity considering their large collections of cultivars, traditional varieties and wild relatives of many crops. This genetic potential can be accessed for identification of novel genes and/or their functional alleles. Several agriculturally relevant genes were obtained from wild relatives and landraces, such as the bacterial blight resistance gene *Xa21* from *O. longistaminata*^28^ and various blast resistance genes such as *Pi9* from *O. minuta*^29^, *Pid3-A4* from *O. rufipogon*^30^ and *Pi-hk1* from a landrace Heikezijing^31^. The available information on molecular markers together with advancement in sequencing technologies can be applied to dissect the molecular diversity of any favourable trait. Sequence based allele mining is one such approach^32^ for exploring allelic diversity of major genes controlling important traits such as disease resistance^23^.

Here we report the allelic diversity of a major rice blast resistance gene *Pi54* in Indian germplasm. *Pi54* (formerly known as *Pi-k*^*h*^) was first identified in the Indian rice cultivar HR22 and was cloned from the indica type rice cultivar Tetep. The gene confers broad-spectrum resistance against Indian rice blast isolates^33^ and is being effectively used in several rice breeding programs in India. *Pi54* belongs to the ‘Coiled Coil - Nucleotide Binding Site - Leucine Rich Repeats’ (CC-NBS-LRR) class of *R* genes and the protein activates several downstream defence related pathways upon pathogen attack^34^. The new *Pi54* alleles that we have identified expand the known *Pi54* allelic series and could potentially serve as a genetic resource for breeding or engineering resistance to rice blast.

## Results

### Selection of rice genotypes for *Pi54* allele mining

**Figure 1:**
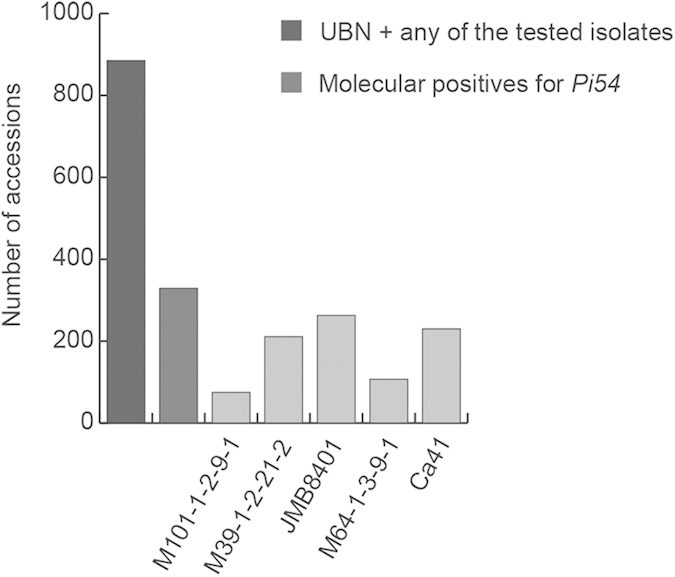
Selection of rice accessions for *Pi54* allele mining. The 885 rice accessions that were resistant with a phenotypic score between 0 and 3 in the uniform blast nursery (UBN) as well as against any of the five pure blast isolates tested^35^ were screened using the Pi54MAS marker. The 329 rice accessions identified as molecular positives were selected as candidates for *Pi54* allele mining. The data on number of accessions resistant (score 0–3) against each of the five blast isolates is also presented.

A set of 885 rice accessions originating from India and found resistant against rice blast, both under natural as well as controlled conditions, were chosen to study the allelic variation of the *Pi54* gene (Fig. 1). These accessions were recorded with a resistant phenotypic score of 0-3 against a field mixture of naturally existing *M. oryzae* strains when screened in the uniform blast nursery (UBN). Under controlled conditions, the selected accessions were found resistant against at least one of the five tested individual blast strains^35^ i.e., M101-1-2-9-1, M39-1-2-21-2, JMB8401, M64-1-3-9-1 and Ca41 (Fig. 1). The 885 Indian accessions are also annotated as blast resistant in the International Rice Germplasm Collection Information System (IRGCIS) of the International Rice Genebank (IRG). The selected accessions represent three major varietal groups, indica (87.9%), javanica (8.6%) and japonica (3.5%). They were further screened for the presence of *Pi54* using the ‘*Pi54* MAS’ marker^36^, which is a functional co-dominant marker that amplifies a 216 bp fragment if a *Pi54* allele is present or a 359 bp fragment if a *Pi54* allele is interrupted by a 144 bp insert (often associated with the susceptible phenotype)^36^ (Supplementary Fig. S1). A total of 329 accessions showed the presence of the amplified 216 bp product and were selected as the candidates for sequence-based allele mining of *Pi54* (Fig. 1, Supplementary Table S1).

### Isolation of new allelic forms of *Pi54*

**Figure 2:**
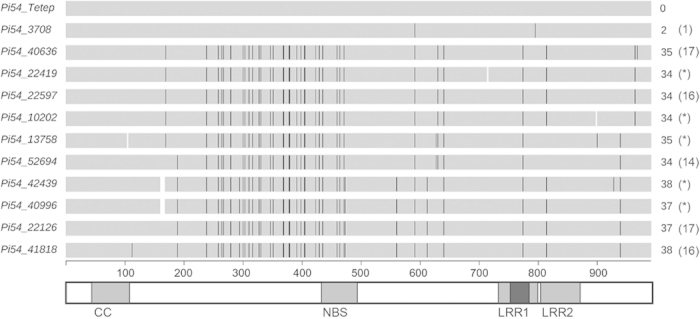
Schematic representation of sequence alignment of newly identified *Pi54* alleles. The DNA sequence alignment of 11 allelic forms of *Pi54* isolated from the studied Indian accessions together with *Pi54_Tetep* as reference is shown. The alleles *Pi54_3708* and *Pi54_40636* were found identical to recently reported alleles of *Pi54*^39^ but the remaining nine alleles are unique. The domain regions of the *Pi54* alleles are illustrated at the bottom as grey boxes (CC, NBS and LRR) and the brown box within LRR1 represents a zinc finger motif. The unit scale indicates the nucleotide position. The black lines on the bars as well as the numbers on the right indicate nucleotide polymorphisms compared to *Pi54_Tetep*. Numbers within brackets indicate the non-synonymous SNPs. The gap in the bars for certain alleles indicates 2 bp (*Pi54_22419*, *Pi54_10202* and *Pi54_13758*) and 8 bp (*Pi54_42439* and *Pi54_40996*) deletions. * indicates the alleles for which non-synonymous SNPs were not calculated due to the presence of deletions causing premature stop codons or a change in the ORF.

Following the confirmation using the diagnostic marker ‘*Pi54* MAS’, the full length coding region of the gene was amplified from all 329 accessions. The primers flanking the start and stop codons as annotated for *Pi54* allele from the cultivar Tetep^37^ (hereafter, referred as *Pi54_Tetep*) were used for the sequence based amplification of *Pi54* alleles. Detailed analysis of the sequences obtained from these 329 accessions identified eleven alleles of *Pi54,* based on the comparison with *Pi54_Tetep*^37^ which was also used as a reference sequence to assign domains. The obtained sequences were further compared with already reported *Pi54* allelic sequences^4^,^38^,^39^,^40^ specifically for the ORF region as reported for *Pi54_Tetep*. The allele *Pi54_3708* and the previously reported *Pi54* alleles from a landrace Satti and the wild rice *O. nivara* were found identical. Similarly *Pi54_40636* was found identical to alleles in the haplogroup-H2 from recently reported *Pi54* alleles^39^. The nine new alleles we identified are unique due to the presence of unique SNPs/deletions and/or combination of shared SNPs observed in these alleles (Supplementary Table S2). Figure 2 shows the schematic representation of the sequence alignment of the identified *Pi54* alleles to the reference allele *Pi54_Tetep*. Except for the three alleles *Pi54_42439*, *Pi54_22419* and *Pi54_3708* that were exclusively identified in single accessions, each of the remaining eight alleles was identified in several rice accessions (Table 1, Supplementary Table S2). *Pi54_40996* was identified in 110 rice accessions and therefore is the most widespread *Pi54* allele. The alleles *Pi54_13758*, *Pi54_22126*, *Pi54_22597* and *Pi54_40636* were also relatively widespread and present in the range of 34 to 53 rice accessions. In addition to the new alleles, 33 accessions have the *Pi54_Tetep* allele (Supplementary Table S2).

### Sequence analysis of *Pi54* alleles

**Figure 3:**
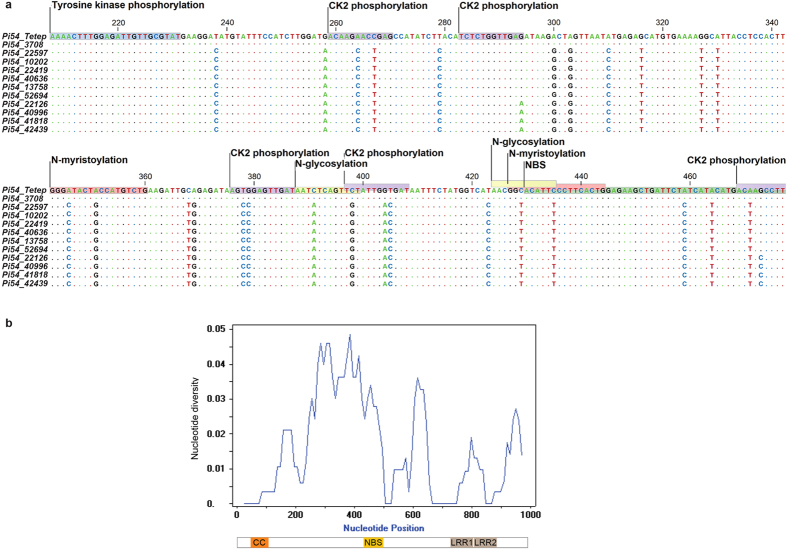
SNP rich region in the new *Pi54* alleles. (**a**) Selection window of the SNP-rich region (between nucleotide position 232 and 429) from the sequence alignment of the eleven studied *Pi54* alleles. Dots represent the nucleotides that are identical to the *Pi54-Tetep* reference and the polymorphisms are represented by single letter code for nucleotides in different colours A (green), T (red), G (black) and C (blue) respectively. The domains and PTM sites are illustrated on top of the reference sequence. The unit scale on top indicates the nucleotide position. (**b**) Sliding-window analysis of nucleotide diversity (π) observed in the identified *Pi54* alleles. The domains are illustrated below the unit scale that represents nucleotide position.

The three major CC, NBS and LRR domains are conserved in the eleven *Pi54* alleles identified in the Indian accessions. Six alleles (*Pi54_22126*, *Pi54_22597*, *Pi54_40636*, *Pi54_41818*, *Pi54_52694* and *Pi54_3708*) have a complete open reading frame (ORF) similar to that of *Pi54_Tetep*. The remaining 5 alleles (*Pi54_40996*, *Pi54_13758*, *Pi54_10202*, *Pi54_42439* and *Pi54_22419*) appear to have shorter ORFs of variable sizes due to predicted premature stop codon(s) introduced by deletions of either 2 bp (*Pi54_22419, Pi54_10202* and *Pi54_13758*) or 8 bp (*Pi54_42439* and *Pi54_40996*) (Fig. 2, Table 1). These eleven alleles differed from *Pi54_Tetep* by a total of 46 nucleotide polymorphisms, three double deletions and a stretch of 8 bp deletions present either uniquely or shared among the different alleles. *Pi54_13758* is the only allele with a double deletion in the CC domain. A ‘SNP-rich region’ with nearly half of the total polymorphisms is found between nucleotides 232 and 429 (Fig. 3a). Five SNPs are present in the NBS domain of which three are synonymous changes (Fig. 2). In case of the LRR domains, one synonymous mutation was found in the Zn finger motif of the LRR1 domain and two non-synonymous mutations were found in both LRR domains, one in each (Fig. 2). All *Pi54* alleles have an intact and highly conserved Zinc finger motif with only a single synonymous mutation at position 774. Similarly, the tyrosine kinase phosphorylation site is highly conserved, suggesting the significance of these motifs for *Pi54* function. In addition, several post-translational modification (PTM) sites such as 13 casein kinase II (CK2) phosphorylation sites, four N-glycosylation sites, four N-myristoylation sites and three protein kinase C (PKC) phosphorylation sites were predicted in all *Pi54* alleles^41^, but their number varied among the alleles because of SNPs and deletions (Table 2). Twenty-nine of the 46 SNPs are shared among all of the alleles except *Pi54_3708*, which differs from *Pi54_Tetep* by only 2 SNPs at positions 591 and 795. One of the two SNPs in *Pi54_3708* is a synonymous mutation at position 591 that is shared with all other identified *Pi54* alleles (i.e., from AAG to AAA; coding for Lysine). Seven alleles have at least one unique SNP (four of them non-synonymous) or deletions specific to that allele but absent in the other *Pi54* alleles (Table 1). The remaining four alleles share the SNPs with the other alleles but in varied combinations that still makes them unique *Pi54* alleles. When compared to the recently reported *Pi54* alleles, 84% of the polymorphisms observed in these eleven alleles were found to be shared with several other *Pi54* alleles, the majority of which were identified in cultivars and landraces reported to be resistant against the diagnostic blast isolate Mo-nwi-37-1^39^. Additionally, several large insertions/deletions were found in recently reported *Pi54* alleles^39^, particularly in those identified from wild rice species, but none of these INDELs were present in the eleven alleles we identified. The presence of these polymorphic yet shared nucleotide changes between alleles indicate frequent sequence exchange between the alleles, possibly by gene conversion.

Nucleotide polymorphism analyses for the eleven *Pi54* alleles were performed in DnaSP^42^. The overall nucleotide diversity (π) of the identified *Pi54* alleles was calculated as 0.01551. The sliding window analysis of nucleotide diversity of *Pi54* alleles showed a high rate of diversity within the SNP-rich region described above (Fig. 3b). The number of mutations (η-46) and the number of segregations sites (S-46) were the same, suggesting their positive selection. The negative Tajima’s D test value (−0.02431) also suggests that the alleles are under positive selection. However, the ratio of non-synonymous to synonymous substitutions per respective site (k_a_/k_s_ value = 0.257) does not indicate a strong positive selection.

### Analysis of predicted Pi54 proteins

**Figure 4:**
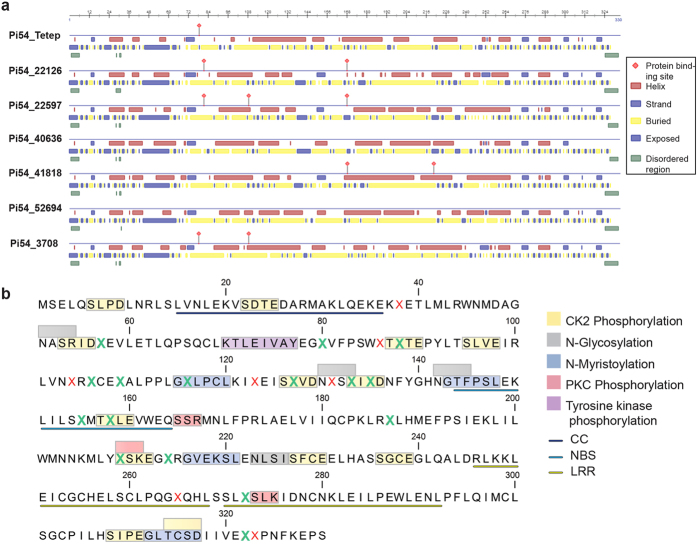
Schematic representation of secondary structure elements, protein-binding sites, exposed/buried and disordered regions within Pi54 proteins. (**a**) Protein sequences of alleles with complete ORFs as that of reference sequence *Pi54_Tetep* were analyzed using ‘PredictProtein’ server. (**b**) Surface accessibility of AA residues of Pi54 proteins predicted using NetSurfP server. ‘X’ represents polymorphic AA residues identified at least in one of the analysed Pi54 proteins. The conserved AA residues are represented by their respective single letter code. The polymorphic residues predicted as solvent exposed are highlighted in green and the ones predicted as buried are in red. The posttranslational modification sites and domain regions are illustrated.

The alleles with a complete ORF based on the *Pi54_Tetep* reference allele were subjected to protein prediction and analyses. Pi54_Tetep has a NBS domain of 21 amino acids (AA) and two non-synonymous mutations in the NBS domain were found among the studied *Pi54* alleles. One of these non-synonymous mutations replaces tyrosine with phenylalanine at position 155 in all predicted proteins except Pi54_3708. The second non-synonymous mutation changes a candidate serine casein kinase II phosphorylation site at position 158 to threonine in Pi54_41818, Pi54_22126, Pi54_40996 and Pi54_42439. Pi54_Tetep has two LRR domains, each 23 AA in length. The only non-synonymous mutation in LRR1 that replaces leucine with phenylalanine at position 265 is observed in Pi54_3708 exclusively (Supplementary Table S3). The non-synonymous mutation in LRR2 changes threonine to alanine at position 272 and is shared among seven predicted Pi54 protein sequences (Pi54_10202, Pi54_40636, Pi54_22597, Pi54_41818, Pi54_22126, Pi54_40996, and Pi54_42439). The allele *Pi54_22419* also has this LRR2-specific mutation but the predicted protein was not analysed because of a two bp deletion in its nucleotide sequence. The ‘PredictProtein’ server^43^ was used to analyse the Pi54 protein sequences of alleles with complete ORFs for secondary structure elements, disordered regions, as well as exposed and buried regions. All analysed Pi54 proteins have predicted disordered regions within their CC domains from position 15 to 36 (Fig. 4a). To predict the surface accessibility of polymorphic AA residues, the Pi54 proteins were subjected to NetSurfP analysis^44^. The majority of the polymorphic residues are exposed and more than half of the predicted exposed residues are located within a PTM site and/or within a specific domain of Pi54 (Fig. 4b) suggesting that these AA changes are likely critical for interaction with *M. oryzae* effector proteins. A single SNP that replaces methionine with leucine at AA position 214 in 10 out of 11 alleles (except *Pi54_3708*) results in a predicted imperfect leucine zipper-like pattern (LxxxxxxLxxxxxxLxxxxxxL) but the predicted protein structures of various alleles retain a typical horse shoe-shaped conformation (Supplementary Fig. S2).

### Phylogeny of new *Pi54* alleles and their distribution among rice subspecies

Five of the eleven *Pi54* alleles are unique to either indica or javanica subspecies, whereas the remaining alleles (*Pi54_40996*, *Pi54_13758*, *Pi54_22126*, *Pi54_22597* and *Pi54_40636*) are distributed among all three subspecies (Table 2). *Pi54_3708* identified in an indica accession in the present study was previously isolated from a landrace Satti as well as from *O. nivara*. *Pi54_10202*, *Pi54_41818*, *Pi54_52694* and *Pi54_42439* were the alleles identified only in the indica subgroup. *Pi54_22419* is the only allele found exclusively in javanica while no allele sequence specific to japonica was identified. The reference allele *Pi54_Tetep* is widespread among indica accessions and is also identified in some of the javanica accessions.

**Figure 5:**
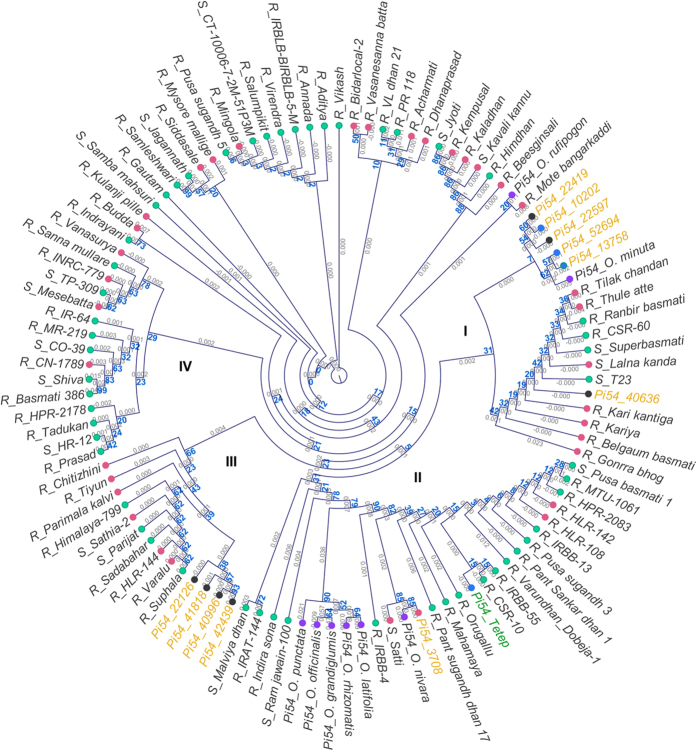
Phylogenetic relationship among *Pi54* alleles. Analysis was performed using *Pi54* sequences identified in our study material together with previously reported *Pi54* sequences from wild rice species and cultivars. The ORF for all the sequences (including the previously reported sequences) were extracted from NCBI database based on the reported ORF for *Pi54_Tetep* reference sequence. Bootstrap values (1000 replications) are mentioned at the branch nodes. The alleles identified in our study material are labelled in yellow and the reference allele in green. The leaf nodes are coloured purple for wild rice, pink for landraces, green for cultivars, black for unknown status or unknown + breeding lines and blue for landraces + unknown/breeding line/cultivar, respectively.

To understand the genetic relatedness among the *Pi54* alleles, phylogeny of the eleven alleles was analyzed together with the previously reported allelic forms of *Pi54,* including the alleles from eight wild rice species^4^,^38^,^40^ and the recently reported *Pi54* allelic sequences from Indian landraces and cultivated varieties^39^. A phylogenetic tree was constructed using the nucleotide sequences corresponding to the complete ORF reported for *Pi54_Tetep*. Four major clusters were observed and the *Pi54* alleles from most of the wild rice accessions formed a separate sub-cluster within cluster II (Fig. 5), but the alleles from *O. nivara*, *O. rufipogon* and *O. minuta* clustered together with alleles from cultivated rice. *Pi54_3708* clustered together with alleles from wild rice *O. nivara*, landrace Satti and other cultivars including *Pi54_Tetep* (Fig. 5) within cluster II. The allele *Pi54_40636* clustered with alleles from landraces and other cultivated rice accessions within cluster I. Except for *Pi54_3708* and *Pi54_40636*, all the other alleles identified in this study formed two clear sub-clusters within cluster I and III. The five alleles in cluster I are closely related to *O. minuta* while the four alleles falling into cluster III show relatedness to different landraces and cultivars.

## Discussion

Considering the narrow genetic base of agriculturally grown varieties, exploration of natural genetic diversity is critically important to broaden and diversify our resistance sources against detrimental crop diseases, including rice blast. Allelic variants of an *R* gene may vary in their *Avr* recognition specificities, thereby conferring race-specific or broad spectrum resistances. For example, assessment of allelic diversity of the barley powdery mildew resistance gene *Mla*^45^ and flax rust resistance gene *L*^46^,^47^ revealed different functional allelic forms carrying several sites of positive selection. The wheat powdery mildew resistance gene *Pm3* currently has 17 identified functional alleles, representing one of the largest allelic series characterized among plant *R* genes^24^. Such allelic variants with SNPs of additive effects are sometimes more rewarding as they might confer broad-spectrum resistance to pathogen isolates from different geographical locations. For example, the orthologue of the rice blast resistance gene *Pid3* isolated from the Indian cultivar Kasalath confers broad-spectrum resistance against Chinese blast isolates (20 of 23 tested isolates) compared to *Pid3* orthologues from other cultivated and wild rice varieties^6^. Similarly, identification of new allelic forms of cloned rice blast *R* genes revealed a unique spectrum of resistance among these alleles, including alleles of *Pi35*^5^, alleles of *Pi54* from *O. rhizomatis* (*Pi54rh*) and *O. officinalis* (*Pi54of*)^4^,^38^, and various alleles at the *AC134922* locus^18^. Notably, *Pi35* that confers quantitative broad-spectrum resistance is an allele of *Pish*, which confers race-specific resistance against rice blast^5^. Here, we investigated the allelic diversity of *Pi54*, a major blast resistance gene conferring resistance against rice blast in India. We identified nine new alleles of *Pi54* that significantly extend the known *Pi54* allelic series. Among the investigated Indian accessions, the distribution of new *Pi54* alleles appeared to be enriched in indica as compared to japonica and javanica accessions.

Pi54 has a NBS-LRR structure with a CC domain at the N terminus^48^. The NBS domain is reported to function as a molecular switch in disease signalling and the LRR region is known to be involved in physical interactions with the AVR proteins^49^. Recent studies also revealed interactions of non-LRR and non-NBS regions of resistance proteins, such as CC domains, with their corresponding AVR proteins^50^,^51^,^52^. The CC domain of the potato Rx protein, which confers resistance against potato virus X, contains disordered regions that engage in intramolecular interaction with the NB-LRR region and intermolecular interaction with the Rx cofactor^53^. Our analysis of Pi54 proteins revealed disordered regions in the CC domain of all alleles, suggesting an important role of the CC domain for the functional structure of Pi54 and in intramolecular and/or intermolecular interactions. The proteins encoded by other allelic forms of *Pi54* (*Pi54*, *Pi54rh*, *Pi54of*) interact with AVR-Pi54 using various domain as well as non-domain regions^4^. These studies together with our own results signify the role of non-domain regions in specificity determination. We have identified only a few polymorphic sites in the NBS and LRR domains of the new *Pi54* alleles, while the majority of the sequence polymorphisms are in the region between the tyrosine kinase phosphorylation site and the NBS domain, which we refer to as the ‘SNP-rich region’. The SNP-rich region as well as the overall nucleotide polymorphism of the alleles identified in this study is shared among the fifty *Pi54* haplotypes that were recently reported from Indian rice varieties^39^. Despite the presence of large InDels in some alleles, such highly conserved polymorphic nucleotides suggest frequent re-shuffling between *Pi54* alleles by gene conversion and recombination.

We found that 67% of the amino acid changes among the minor motifs/patterns and PTM sites are also in the SNP-rich region of the gene. The majority of the polymorphisms at the PTM sites does not result in their loss but could influence the accessibility of acceptor residue(s) within a PTM site for the modifying enzymes. More than half of the polymorphic AA residues predicted as exposed are located within a PTM site. It has been suggested that the difference in number and distribution of phosphorylation motifs between the resistant Tetep and susceptible Nipponbare alleles of *Pi54* are responsible for the resistance phenotype in Tetep^54^. We therefore expect that the observed changes in the PTM sites together with the shared and unique SNPs observed (in domain and non-domain regions) among the identified alleles might influence the protein structure, their interactions with effector proteins and downstream defence signalling. This in turn could result in novel resistance specificities. The varied patterns of resistance/susceptibility among the rice accessions containing the new *Pi54* alleles to different rice blast strains further indicate a possibility of novel and/or altered resistance spectrum as compared to the known *Pi54* alleles (Supplementary Table S1). However, this would need to be functionally tested using approaches such as gene silencing and/or complementation assays. Although the presented alleles were isolated from selected rice accessions that are annotated as blast resistant in IRGCIS as well as in our own screening experiments under natural and controlled conditions, the possibility for resistance in these lines due to the presence of other major *R* gene(s) and/or QTLs cannot be ruled out. Once functionally validated, these alleles could be used in various combinations with previously reported allelic forms of *Pi54* or with other rice blast resistance genes in order to enhance the durability of blast resistance. Furthermore, detailed understanding of polymorphic sites controlling resistance will facilitate intragenic allele pyramiding to combine resistance specificities of different alleles^25^.

## Materials and Methods

### Plant material and molecular screening for *Pi54*

The rice germplasm material for this study was obtained from the International Rice Genebank (IRG) of the International Rice Research Institute (IRRI), Philippines. The accessions were screened for rice blast resistance against field mix-inoculum and five pure blast isolates^35^. Based on the standard scoring system for leaf blast^55^ (scale 0–9), accessions that were resistant with a phenotypic score of 0-3 against field mix-inoculum and against any of the five isolates were selected for molecular screening. Molecular screening was carried out to identify the presence of *Pi54* using a functional co-dominant marker ‘*Pi54* MAS’^36^ that differentiates *Pi54* alleles with and without an insertion of 144 bp, the former being correlated with the susceptible allele. Genomic DNA extracted from *Pi54* monogenic line was used as the positive control and that from susceptible rice cultivar LTH was used as the negative control for the Polymerase chain reaction (PCR), in addition to a water control.

### Isolation and cloning of *Pi54* alleles

Forward primer 5′-TACCTGATGGTTCTTTAAAATTGGG (designed for this study) and reverse primer 5′-CATAAGCTAGACCTTGAAGGATGTC^38^ were used for isolation of the full-length coding region of *Pi54* alleles as annotated for *Pi54* allele from cultivar Tetep^37^. PCR was performed with an initial denaturation at 95 °C for 5 minutes; followed by additional denaturation at 98 °C for 20 seconds, annealing at 61 °C for 20 seconds, extension at 72 °C for 1 minute (these three steps repeated for 35 cycles); followed by final extension at 72 °C for 2 minutes, using KAPA HiFi HotStart DNA polymerase (high fidelity proof-reading enzyme). The amplified products were cloned using pJET1.2 blunt end cloning vector.

### Sequencing and sequence analysis

Forward primer 5′-GTTAGGCCTTCAGGAATGGAGTGC was used as internal sequence primer in addition to the standard forward and reverse primers from the pJET1.2 cloning vector for the complete sequence coverage. The primers for isolation and sequencing of *Pi54* alleles were designed in DNASTAR-SeqBuilder, using *Pi54_Tetep* as reference (Accession Nr. AY914077). DNA sequencing was performed using Applied Biosystems Capillary Sequencer 3730. Sequence assembly, consensus development and alignments were done using ‘CLC Genomics Workbench, version-7.5′. Multiple sequence alignment was performed to identify the Single Nucleotide Polymorphisms (SNPs). Sequence polymorphism analyses such as, number of segregation sites and mutations, sliding window analysis of nucleotide diversity, k_a_/k_s_ ratio and Tajima’s D test were performed using DnaSP-v5^42^. Alleles with unique SNPs or InDels (insertion or deletion) were re-sequenced and in addition, re-amplified (from genomic DNA of respective accessions), re-cloned and re-sequenced for SNP/InDel confirmation. For the allelic forms identified in more than four accessions (thereby representing multiple independent amplification and cloning events), no repetitions were made.

### Phylogenetic analysis

Phylogenetic analysis was performed using DNA sequences of the newly identified *Pi54* alleles together with previously reported *Pi54* alleles from cultivated rice, landraces and wild rice species to analyse their genetic relatedness^4^,^38^,^39^,^40^. The nucleotide sequences of previously reported *Pi54* alleles i.e., from start to stop codon (including introns, if any) corresponding to that of *Pi54*_*Tetep* were obtained from NCBI. The sequence alignments were performed in CLC genomics workbench. The tree was constructed using ‘Neighbor-joining’ method and ‘Jukes-Cantor’ for nucleotide distance measure and bootstrap performed with 1000 replications.

### Protein prediction and analysis

The protein sequences of *Pi54* alleles were obtained using the EXPASY translation tool (http://web.expasy.org/translate/). The major domains such as NBS and LRR regions were assigned as previously reported for *Pi54*^37^,^40^. The CC domain was predicted using the COILS server (http://www.ch.embnet.org/software/COILS_form.html). The patterns and post-translational modification sites were predicted using ScanProsite server^41^. The secondary structure, solvent accessibility, disorders and flexibility regions of the predicted proteins were analysed using the PredictProtein server^43^. The surface accessibility of AA residues of Pi54 proteins were predicted in NetSurfP server^44^. Phyre2 server^56^ was used for three-dimensional protein structure predictions of proteins of the alleles with complete ORFs based on the *Pi54_Tetep* reference allele. The prediction parameter was set to ‘normal’ mode for all the proteins analysed. PyMOL-version 1.3 was used to visualize, analyse and edit the protein structures for their secondary structure elements and orientation.

## Additional Information

**Accession codes:** The newly identified Pi54 sequences have been submitted to the GenBank database with accession numbers, Pi54_40996 = KR052922, Pi54_13758 = KR052923, Pi54_22126 = KR052924, Pi54_22597 = KR052925, Pi54_40636 = KR052926, Pi54_10202 = KR052927, Pi54_41818 = KR052928, Pi54_52694 = KR052929, Pi54_42439 = KR052930, Pi54_22419 = KR052931, and Pi54_3708 = KR052932.

**How to cite this article**: Vasudevan, K. *et al.* Identification of novel alleles of the rice blast resistance gene *Pi54*. *Sci. Rep.* **5**, 15678; doi: 10.1038/srep15678 (2015).

## Supplementary Material

Supplementary Information

## Figures and Tables

**Table 1:** Sequence characteristics of *Pi54* alleles and their distribution among rice varietal groups.

*Pi54* allele	Number of accessions carrying the allele	%identity to *Pi54_Tetep*	Number of SNP sites (non-synonymous)	Unique SNPs/InDels	Deletions	Number of accessions belonging to a particular sub-species		
						Indica	Japonica	Javanica
*Pi54_40996*	110	95.46	37 (*)	–	8 bp	108	1	1
*Pi54_13758*	53	96.27	35 (*)	Yes	2 bp	30	6	17
*Pi54_22126*	42	96.27	37 (17)	–	No	35	2	5
*Pi54_22597*	39	96.57	34 (17)	–	No	16	2	21
*Pi54_40636*	34	96.47	35 (18)	Yes	No	33	1	–
*Pi54_10202*	8	96.37	34 (*)	Yes	2 bp	8	–	–
*Pi54_41818*	4	96.17	38 (18)	Yes	No	4	–	–
*Pi54_52694*	3	96.57	34 (15)	—	No	3	–	–
*Pi54_42439*	1	95.36	38 (*)	Yes	8 bp	1	–	–
*Pi54_22419*	1	96.37	34 (*)	Yes	2 bp	–	–	1
*Pi54_3708*	1	99	2 (1)	Yes	No	1	-	–
*Pi54_Tetep*	33	–	–	–	–	30	–	3

^*^Non-synonymous SNPs for these alleles were not calculated, as there are predicted premature stop codon(s) or changes in ORF due to 2 or 8 bp deletions in these alleles. Two of the eleven alleles identified in this study material i.e., *Pi54_3708* and *Pi54_40636* are found identical to already known *Pi54* sequences.

**Table 2:** Number of various motifs/posttranslational modification sites observed in *Pi54* alleles.

Pi54 protein	Tyrosine kinase phosphorylation site	Casein kinase II phosphorylation site	N-glycosylation site	N-myristoylation site	Protein kinase C phosphorylation site	Leucine zipper-like pattern
Pi54_Tetep	1	13	4	4	3	–
Pi54_40996	1	10	3	4	3	1
Pi54_13758	1	10	4	4	2	1
Pi54_22126	1	13	4	4	3	1
Pi54_22597	1	13	4	4	3	1
Pi54_40636	1	13	4	4	3	1
Pi54_10202	1	11	4	3	3	1
Pi54_41818	1	13	4	4	3	1
Pi54_52694	1	12	4	4	2	1
Pi54_42439	1	10	3	4	3	1
Pi54_22419	1	10	4	3	2	1
Pi54_3708	1	13	4	4	3	–

The predicted Pi54 protein sequences were analysed for the presence of different motifs using ScanProsite server.
