# Corrigendum: Identification of novel alleles of the rice blast resistance gene *Pi54*

**DOI:** 10.1038/srep17920

**Published:** 2016-01-06

**Authors:** Kumar Vasudevan, Wilhelm Gruissem, Navreet K. Bhullar

Scientific Reports 5: Article number: 15678; 10.1038/srep15678published online: 10262015; updated: 01062016

There is an error in the Introduction of this Article.

"*Pi54* (formerly known as *Pi-k*^*h*^) was first identified in the Indian rice cultivar HR22 and was cloned from the indica type rice cultivar Tetep."

should read:

"Pi54 was cloned from the indica type rice cultivar Tetep."

