# Supplementary material for: Identification of novel alleles of the rice blast resistance gene Pi54
Source: Sci Rep. 2015 Oct 26;5:15678. doi: 10.1038/srep15678 (PMC4620502; doi:10.1038/srep15678)
Supplement: Supplementary Information [file srep15678-s1.pdf]

Identification of novel alleles of the rice blast resistance gene *Pi54*

Authors and affiliation: Kumar Vasudevan, Wilhelm Gruissem and Navreet K. Bhullar*

Plant Biotechnology, Department of Biology, ETH Zurich (Swiss Federal Institute of Technology), Zurich, Switzerland

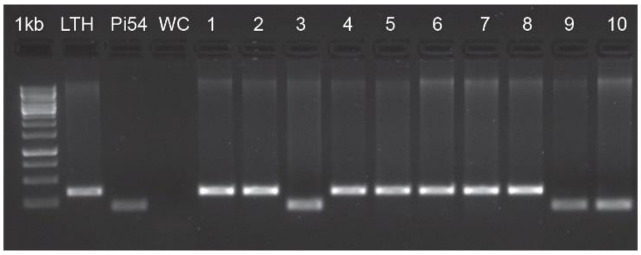

Supplementary Figure S1. Sample picture of molecular screening for *Pi54*

Molecular screening was performed using the co-dominant marker *Pi54MAS*. 1kb, DNA marker; LTH, negative control; *Pi54*, positive control (*Pi54*-monogenic line); WC, water control; 1–10, test accessions. The accessions that show the amplified product of a 216 bp fragment i.e., 3 (IRGC-42412), 9 (IRGC-42422), and 10 (IRGC-42423) represent the presence of *Pi54* allele and that of a 359 bp fragment i.e., 1 (IRGC-42403), 2 (IRGC-42405), 4 (IRGC-42413), 5 (IRGC-42417), 6 (IRGC-42418), 7 (IRGC-42419) and 8 (IRGC-42420) represent the presence of *Pi54* allele interrupted by a 144 bp insertion (often associated with the susceptible phenotype)[36].

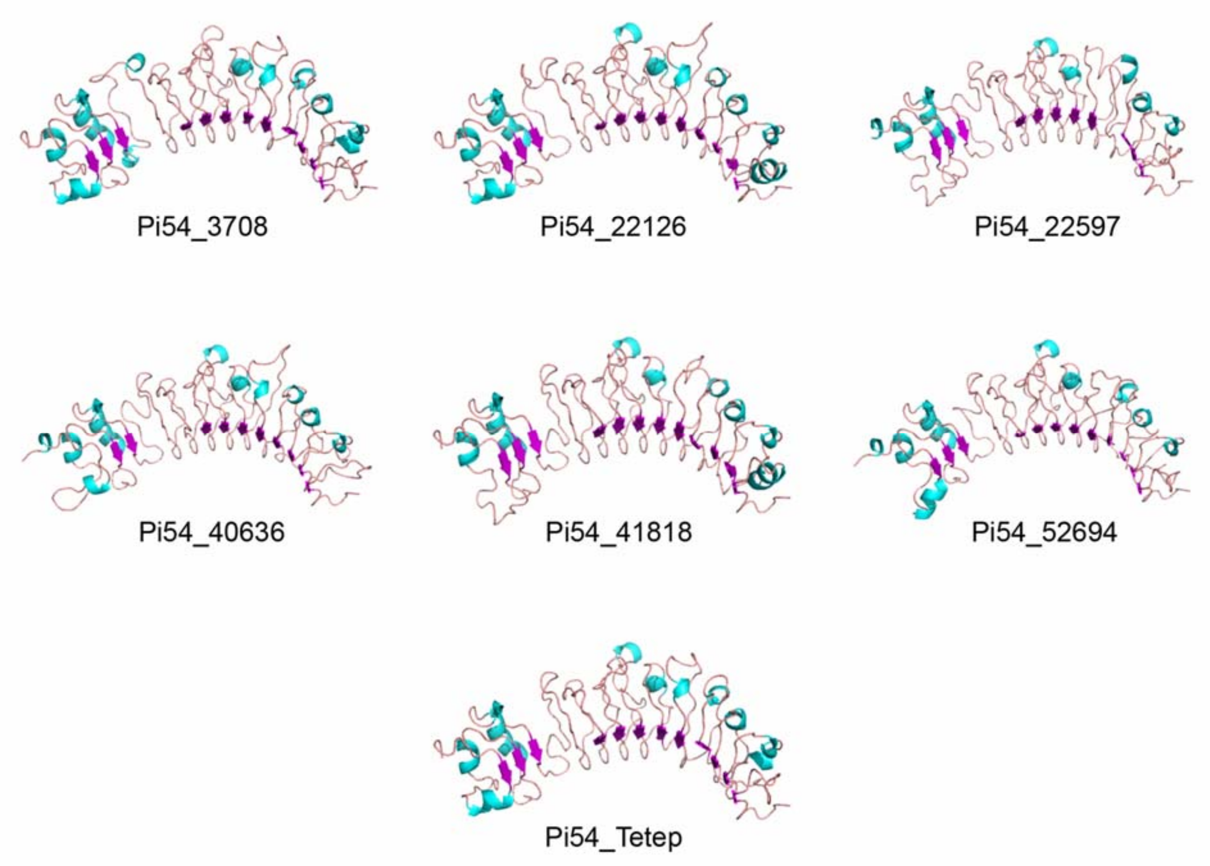

Supplementary Figure S2. Predicted three-dimensional structures of Pi54 proteins

The protein 3D structures of *Pi54* alleles with a complete ORF are presented. The structures were predicted using the Phyre2 server in prediction mode-normal. All the protein structures are presented at standard orientation using PyMOL, version-1.3. The secondary structures elements are presented in different colour i.e., helix (cyan), sheet (magenta) and loop (light-orange).

Supplementary Table S2. List of IRGC accessions carrying the respective *Pi54* alleles identified in this study.

Alleles identified in this study have been listed after the IRGC accession from which the respective allele was first isolated. The accessions chosen to represent the respective allele of *Pi54* are indicated in red.

***Pi54* alleles**	**IRGC accessions carrying the respective *Pi54* alleles**
Pi54_40996	6113; 12170; 12248; 19310; 19313; 19314; 19568; 20283; 21003; 21045; 21059; 21062; 21065; 21148; 21701; 21955; 22071; 22179; 24243; 24256; 24258; 40996; 41005; 41010; 41014; 41015; 41075; 41084; 41117; 41142; 41150; 41195; 41217; 41233; 41283; 41285; 41294; 41297; 41299; 41305; 41307; 41308; 41311; 41325; 41326; 41327; 41328; 41356; 41364; 41368; 41375; 41395; 41508; 41535; 41539; 41582; 41615; 41616; 41617; 41620; 41625; 41628; 41633; 41655; 41690; 41699; 41732; 1964; 42116; 42158; 42165; 42172; 42173; 42220; 42221; 42245; 42263; 42264; 42293; 42307; 42308; 42315; 42319; 42321; 42326; 42339; 42340; 42383; 42385; 42423; 42425; 42482; 42601; 42676; 42677; 42678; 42679; 42703; 42734; 42792; 42821; 42832; 42833; 42836; 42838; 42844; 42921; 43211; 49729; 49856
Pi54_13758	191; 5794; 5796; 5836; 9807; 13758; 14438; 20277; 20512; 20578; 20588; 20629; 20707; 20761; 20892; 21213; 21273; 21330; 21368; 21381; 21392; 21511; 21562; 21563; 22007; 22020; 22024; 22054; 22055; 22074; 22081; 22088; 22109; 22117; 22162; 22165; 22168; 22240; 22254; 22258; 22273; 22300; 22315; 22318; 22320; 22381; 22512; 22518; 22522; 22525; 24266; 42014; 42664
Pi54_22126	74; 6304; 14574; 21400; 21414; 21415; 21418; 21467; 21560; 21842; 21866; 22046; 22061; 22067; 22089; 22126; 22132; 22145; 22146; 22160; 22172; 22622; 22653; 22679; 22698; 24270; 41044; 41112; 41831; 41895; 42320; 42335; 42336; 42770; 43035; 45093; 45115; 45212; 47580; 49560; 49561; 49778
Pi54_22597	5791; 6447; 9752; 10620; 20586; 20636; 20671; 20830; 21516; 21753; 21785; 21861; 22045; 22051; 22059; 22065; 22069; 22092; 22326; 22337; 22349; 22363; 22394; 22410; 22422; 22427; 22446; 22542; 22544; 22586; 22593; 22597; 22613; 22702; 22705; 41744; 42922; 45226; 49743
Pi54_40636	673; 3652; 3699; 3703; 3707; 3719; 3750; 3751; 4895; 5056; 6205; 6298; 6394; 10912; 11052; 11062; 14427; 21911; 21917; 22015; 22600; 32559; 40636; 41509; 41736; 41882; 41938; 45151; 45284; 45305; 46066; 49558; 49733; 49850
Pi54_10202	4799; 5971; 10202; 22540; 45272; 46028; 46123; 46879
Pi54_41818	41818; 45037; 45043; 45946
Pi54_52694	36764; 45807; 52694
Pi54_42439	42439
Pi54_22419	22419
Pi54_3708	3708
Pi54_Tetep	3661; 3663; 3700; 3722; 4820; 6264; 10299; 12121; 19379; 19682; 20687; 21537; 21792; 22021; 22039; 22350; 22368; 22369; 22375; 22401; 22409; 22433; 22535; 22614; 41844; 42412; 45310; 45500; 45530; 45852; 46102; 46632; 46837

Supplementary Table S3. Unique SNPs in *Pi54* alleles.
Position is based on reference gene sequence

***Pi54* allele**	**Unique non-synonymous SNPs**	**Effect on protein sequence (AA position)**
Pi54_3708	1 SNP at position 795	Replace leucine with phenylalanine (265) in LRR1
Pi54_41818	1 SNP at position 112	Replace leucine with phenylalanine (38)
Pi54_42439	1 SNP at position 928	Replace proline with serine (260) within one of the CK2 phosphorylation sites
Pi54_40636	1 SNP at position 968	Replace cysteine with phenylalanine (323)

Identification of novel alleles of the rice blast resistance gene *Pi54*

Authors and affiliation: Kumar Vasudevan, Wilhelm Gruissem and Navreet K. Bhullar*

Plant Biotechnology, Department of Biology, ETH Zurich (Swiss Federal Institute of Technology), Zurich, Switzerland

Supplementary Table S1

Detailed information regarding the 329 accessions carrying the *Pi54* allele

IRGC Accession number	Variety name*	Source country*	Varietal group*	Status of sample*	Collection location*	*Pi54* allele category identified	UBN score	M101-1-2-9-1	M39-1-2-21-2	JMB8401	M64-1-3-9-1	Ca41
74	T 1	INDIA	Indica	Released/Improved cultivar	NA	*Pi54_22126*	0	6	6	1	7	3
191	NHTA10	INDIA	Indica		NA	*Pi54_13758*	2	4	1	2	6	5
673	HR22	INDIA	Indica	Breeding and inbred line	NA	*Pi54_40636*	0	6	7	2	8	2
3652	MUSHKAN	INDIA	Indica		NA	*Pi54_40636*	0	6	0	7	6	7
3661	PALMAN SELN	INDIA	Indica	Breeding and inbred line	NA	*Pi54_Tetep*	3	6	-	3	6	2
3663	BANSI	INDIA	Indica	Landrace/Traditional cultivar	NA	*Pi54_Tetep*	0	6	-	3	5	2
3699	N 12	INDIA	Indica	Released/Improved cultivar	NA	*Pi54_40636*	0	8	6	4	5	2
3700	NP97	INDIA	Indica	Breeding and inbred line	NA	*Pi54_Tetep*	0	8	7	3	5	1
3703	NP137	INDIA	Indica	Breeding and inbred line	NA	*Pi54_40636*	0	8	6	7	5	2
3707	ANDI FROM N. POKHARA	INDIA	Indica		NA	*Pi54_40636*	0	2	0	7	1	0
3708	EARLY 3	INDIA	Indica	Landrace/Traditional cultivar	NA	*Pi54_3708*	0	7	1	3	7	5
3719	T 9	INDIA	Indica	Released/Improved cultivar	NA	*Pi54_40636*	0	7	8	7	1	5
3722	T 137	INDIA	Indica	Released/Improved cultivar	NA	*Pi54_Tetep*	0	7	4	1	5	1
3750	BASMATI 370	INDIA	Indica		NA	*Pi54_40636*	0	8	7	7	3	3
3751	MUSKKAN 41	INDIA	Indica		NA	*Pi54_40636*	0	8	7	7	6	1
4799	99216	INDIA	Indica		NA	*Pi54_10202*	0	8	0	8	7	7
4820	T 1	INDIA	Indica		NA	*Pi54_Tetep*	3	8	7	4	4	1
4895	BASMATI 370	INDIA	Indica		Punjab	*Pi54_40636*	0	7	0	7	3	2
5056	NP130	INDIA	Indica	Breeding and inbred line	NA	*Pi54_40636*	0	7	6	2	7	2
5791	JHUM PADDY 2	INDIA	Indica		NA	*Pi54_22597*	0	7	3	1	1	0
5794	JHUM PADDY 5	INDIA	Indica		NA	*Pi54_13758*	0	5	-	0	-	0
5796	JHUM PADDY 7	INDIA	Indica		NA	*Pi54_13758*	0	6	5	1	4	1
5836	HR59	INDIA	Indica	Breeding and inbred line	NA	*Pi54_13758*	0	5	0	8	4	0
5971	SANNABATHA	INDIA	Indica		NA	*Pi54_10202*	0	5	4	7	3	2
6113	PTB21	INDIA	Indica	Breeding and inbred line	NA	*Pi54_40996*	0	4	4	7	5	1
6205	R 10	INDIA	Indica		NA	*Pi54_40636*	0	7	2	2	6	4
6264	N 22	INDIA	Indica	Breeding and inbred line	NA	*Pi54_Tetep*	0	6	3	1	5	4
6298	N 12	INDIA	Indica	Breeding and inbred line	NA	*Pi54_40636*	0	7	2	4	3	4
6304	PTB30	INDIA	Indica	Breeding and inbred line	NA	*Pi54_22126*	0	8	6	4	5	3
6394	HR22	INDIA	Indica	Breeding and inbred line	NA	*Pi54_40636*	0	8	3	3	6	3
6447	BASMATI T 3	INDIA	Indica	Breeding and inbred line	NA	*Pi54_22597*	0	3	5	3	7	3
9752	JBS438	INDIA	Indica	Breeding and inbred line	NA	*Pi54_22597*	0	8	8	5	8	3
9807	ASM10	INDIA	Indica		NA	*Pi54_13758*	0	1	0	1	4	0
10202	BARO SADDY	INDIA	Indica	Landrace/Traditional cultivar	NA	*Pi54_10202*	0	4	0	5	1	0
10299	TINPAKHIA	INDIA	Indica	Breeding and inbred line	NA	*Pi54_Tetep*	2	7	7	2	8	1
10620	AP439	INDIA	Indica	Breeding and inbred line	NA	*Pi54_22597*	0	5	2	0	1	2
10912	NP130 MUTANT S 1	INDIA	Indica		NA	*Pi54_40636*	0	7	6	1	3	2
11052	PTB18	INDIA	Indica	Breeding and inbred line	NA	*Pi54_40636*	0	4	4	5	5	1
11062	G 378	INDIA	Indica	Breeding and inbred line	NA	*Pi54_40636*	0	8	3	4	3	5
12121	ARC 5754	INDIA	Indica		Assam	*Pi54_Tetep*	3	1	0	5	4	4
12170	ARC 5994	INDIA	Indica		Assam	*Pi54_40996*	0	7	0	1	3	0
12248	ARC 6178	INDIA	Indica		Assam	*Pi54_40996*	1	4	0	0	3	0

13758	MOCHANGRA	INDIA	Indica	Landrace/Traditional cultivar	Manipur	*Pi54_13758*	0	6	3	0	6	1
14427	IARI 5828	INDIA	Japonica		NA	*Pi54_40636*	0	5	1	0	1	2
14438	IARI 10376	INDIA	Indica		NA	*Pi54_13758*	0	4	1	4	3	1
14574	ARC 11443 A	INDIA	Javanica		Arunachal Pradesh	*Pi54_22126*	0	6	1	0	1	0
19310	PTB12	INDIA	Indica	Breeding and inbred line	NA	*Pi54_40996*	0	7	4	3	5	1
19313	PTB20	INDIA	Indica	Breeding and inbred line	NA	*Pi54_40996*	0	8	4	5	5	2
19314	PTB21	INDIA	Indica	Breeding and inbred line	NA	*Pi54_40996*	0	4	6	1	1	1
19379	N 22	INDIA	Indica		NA	*Pi54_Tetep*	0	4	5	1	7	3
19568	IARI 10840	INDIA	Indica	Released/Improved cultivar	NA	*Pi54_40996*	0	6	6	1	7	3
19682	METTASANNALU	INDIA	Indica		NA	*Pi54_Tetep*	0	7	1	1	7	0
20277	ARC 5931	INDIA	Indica		Assam	*Pi54_13758*	2	3	2	1	1	0
20283	ARC 5942	INDIA	Indica		Assam	*Pi54_40996*	0	2	4	8	4	5
20512	ARC 7113	INDIA	Indica		Meghalaya	*Pi54_13758*	0	8	8	4	6	2
20578	ARC 7283	INDIA	Indica		Meghalaya	*Pi54_13758*	0	7	7	3	3	1
20586	ARC 7297	INDIA	Indica		Meghalaya	*Pi54_22597*	0	7	7	2	6	2
20588	ARC 7299	INDIA	Japonica		Meghalaya	*Pi54_13758*	1	8	7	-	8	2
20629	ARC 7428	INDIA	Javanica		Meghalaya	*Pi54_13758*	0	8	8	4	8	3
20636	ARC 10008	INDIA	Javanica		Meghalaya	*Pi54_22597*	0	7	6	1	8	2
20671	ARC 10051	INDIA	Javanica		Meghalaya	*Pi54_22597*	0	8	7	1	8	2
20687	ARC 10070	INDIA	Indica		Meghalaya	*Pi54_Tetep*	0	8	7	1	5	5
20707	ARC 10097	INDIA	Indica		Meghalaya	*Pi54_13758*	0	5	6	3	6	4
20761	ARC 10168	INDIA	Indica		Meghalaya	*Pi54_13758*	0	8	5	3	8	1
20830	ARC 10289	INDIA	Indica		Meghalaya	*Pi54_22597*	0	8	5	3	7	1
20892	ARC 10383	INDIA	Indica		Meghalaya	*Pi54_13758*	0	8	6	2	8	5
21003	ARC 10579	INDIA	Indica		Meghalaya	*Pi54_40996*	0	4	0	0	7	1
21045	ARC 10721	INDIA	Indica		Meghalaya	*Pi54_40996*	0	8	0	3	2	1
21059	ARC 10756	INDIA	Indica		Meghalaya	*Pi54_40996*	0	7	3	2	7	1
21062	ARC 10776	INDIA	Indica		Meghalaya	*Pi54_40996*	0	7	1	1	6	3
21065	ARC 10783	INDIA	Indica		Meghalaya	*Pi54_40996*	0	6	0	1	8	3
21148	ARC 10936	INDIA	Indica		Meghalaya	*Pi54_40996*	0	4	0	3	2	1
21213	ARC 11134	INDIA	Indica		Assam	*Pi54_13758*	0	8	7	1	8	6
21273	ARC 11271	INDIA	Javanica		Meghalaya	*Pi54_13758*	0	5	7	1	3	6
21330	ARC 11339	INDIA	Japonica		Meghalaya	*Pi54_13758*	0	3	5	0	5	5
21368	ARC 11381	INDIA	Indica		Arunachal Pradesh	*Pi54_13758*	0	8	5	0	7	2
21381	ARC 11425	INDIA	Javanica		Arunachal Pradesh	*Pi54_13758*	0	4	4	1	1	1
21392	ARC 11440	INDIA	Javanica		Arunachal Pradesh	*Pi54_13758*	0	7	4	1	2	0
21400	ARC 11460	INDIA	Javanica		Arunachal Pradesh	*Pi54_22126*	0	6	1	1	2	1
21414	ARC 11474	INDIA	Japonica		Arunachal Pradesh	*Pi54_22126*	0	1	1	1	1	0
21415	ARC 11475	INDIA	Javanica		Arunachal Pradesh	*Pi54_22126*	0	5	6	1	5	2
21418	ARC 11478	INDIA	Javanica		Arunachal Pradesh	*Pi54_22126*	0	1	6	1	-	0
21467	ARC 11542	INDIA	Indica		Arunachal Pradesh	*Pi54_22126*	0	7	7	1	6	1
21511	ARC 11604	INDIA	Javanica		Arunachal Pradesh	*Pi54_13758*	0	2	0	1	1	0
21516	ARC 11609	INDIA	Javanica		Arunachal Pradesh	*Pi54_22597*	0	2	0	0	4	0
21537	ARC 11639	INDIA	Javanica		Arunachal Pradesh	*Pi54_Tetep*	0	4	1	1	3	0
21560	ARC 11684	INDIA	Javanica		Arunachal Pradesh	*Pi54_22126*	2	7	0	1	3	0
21562	ARC 11686	INDIA	Japonica		Arunachal Pradesh	*Pi54_13758*	0	7	2	0	5	0
21563	ARC 11687	INDIA	Japonica		Arunachal Pradesh	*Pi54_13758*	0	7	4	2	4	1
21701	ARC 11859	INDIA	Indica		Arunachal Pradesh	*Pi54_40996*	0	8	4	2	1	4
21753	ARC 11929	INDIA	Indica		Arunachal Pradesh	*Pi54_22597*	0	6	4	1	1	1
21785	ARC 11964	INDIA	Japonica		Arunachal Pradesh	*Pi54_22597*	0	4	0	0	2	2
21792	ARC 11971	INDIA	Javanica		Arunachal Pradesh	*Pi54_Tetep*	0	5	4	1	6	3

21842	ARC 12027	INDIA	Indica		Arunachal Pradesh	*Pi54_22126*	0	6	5	1	5	1
21861	ARC 12046	INDIA	Indica		Arunachal Pradesh	*Pi54_22597*	0	7	8	1	5	3
21866	ARC 12051	INDIA	Indica		Arunachal Pradesh	*Pi54_22126*	0	1	6	1	5	1
21911	ARC 12105	INDIA	Indica		Arunachal Pradesh	*Pi54_40636*	0	1	2	0	-	1
21917	ARC 12111	INDIA	Indica		Arunachal Pradesh	*Pi54_40636*	0	2	7	4	7	5
21955	ARC 12166	INDIA	Indica		Arunachal Pradesh	*Pi54_40996*	0	4	0	5	4	1
22007	ARC 12324	INDIA	Indica		Arunachal Pradesh	*Pi54_13758*	0	6	5	3	7	1
22015	ARC 12358	INDIA	Indica		Arunachal Pradesh	*Pi54_40636*	0	5	2	1	1	0
22020	ARC 12384	INDIA	Indica		Arunachal Pradesh	*Pi54_13758*	0	2	0	0	8	1
22021	ARC 12385	INDIA	Javanica		Nagaland	*Pi54_Tetep*	1	3	2	0	7	2
22024	ARC 12389	INDIA	Javanica		Nagaland	*Pi54_13758*	0	4	1	0	8	1
22039	ARC 12407	INDIA	Indica		Nagaland	*Pi54_Tetep*	0	6	2	3	3	1
22045	ARC 12414	INDIA	Javanica		Nagaland	*Pi54_22597*	0	1	0	0	1	1
22046	ARC 12415	INDIA	Indica		Nagaland	*Pi54_22126*	0	1	0	0	1	0
22051	ARC 12420	INDIA	Javanica		Nagaland	*Pi54_22597*	0	1	0	1	1	0
22054	ARC 12423	INDIA	Javanica		Nagaland	*Pi54_13758*	0	1	1	0	5	1
22055	ARC 12424	INDIA	Javanica		Nagaland	*Pi54_13758*	0	0	1	0	1	0
22059	ARC 12428	INDIA	Javanica		Nagaland	*Pi54_22597*	0	6	1	1	5	0
22061	ARC 12430	INDIA	Indica		Nagaland	*Pi54_22126*	0	5	4	3	6	1
22065	ARC 12435	INDIA	Indica		Nagaland	*Pi54_22597*	2	6	2	1	5	0
22067	ARC 12437	INDIA	Indica		Nagaland	*Pi54_22126*	0	4	5	1	8	1
22069	ARC 12439	INDIA	Javanica		Nagaland	*Pi54_22597*	0	5	4	0	8	1
22071	ARC 12441	INDIA	Japonica		Nagaland	*Pi54_40996*	0	6	6	0	8	1
22074	ARC 12444	INDIA	Javanica		Arunachal Pradesh	*Pi54_13758*	0	1	0	1	7	1
22081	ARC 12475	INDIA	Javanica		Nagaland	*Pi54_13758*	0	1	0	1	1	0
22088	ARC 12482	INDIA	Javanica		Nagaland	*Pi54_13758*	0	3	1	0	7	3
22089	ARC 12483	INDIA	Indica		Nagaland	*Pi54_22126*	0	7	0	3	6	0
22092	ARC 12487	INDIA	Javanica		Nagaland	*Pi54_22597*	0	3	0	2	1	1
22109	ARC 12504	INDIA	Indica		Nagaland	*Pi54_13758*	0	5	0	0	8	2
22117	ARC 12514	INDIA	Indica		Nagaland	*Pi54_13758*	0	2	0	1	7	1
22126	ARC 12524	INDIA	Indica		Nagaland	*Pi54_22126*	1	6	2	2	3	0
22132	ARC 12539	INDIA	Indica		Nagaland	*Pi54_22126*	0	3	4	2	5	1
22145	ARC 12555	INDIA	Indica		Nagaland	*Pi54_22126*	3	3	2	0	4	3
22146	ARC 12557	INDIA	Indica		Nagaland	*Pi54_22126*	0	1	0	0	1	2
22160	ARC 12571	INDIA	Indica		Nagaland	*Pi54_22126*	0	5	1	2	1	1
22162	ARC 12574	INDIA	Javanica		Nagaland	*Pi54_13758*	0	4	1	1	0	1
22165	ARC 12580	INDIA	Indica		Nagaland	*Pi54_13758*	0	6	0	0	0	3
22168	ARC 12586	INDIA	Indica		Nagaland	*Pi54_13758*	0	8	2	5	5	3
22172	ARC 12590	INDIA	Indica		Nagaland	*Pi54_22126*	0	8	0	4	6	2
22179	ARC 12599	INDIA	Indica		Arunachal Pradesh	*Pi54_40996*	0	5	3	4	7	2
22240	ARC 12669	INDIA	Indica		Arunachal Pradesh	*Pi54_13758*	0	1	1	1	7	2
22254	ARC 12687	INDIA	Javanica		Arunachal Pradesh	*Pi54_13758*	0	2	1	1	8	1
22258	ARC 12691	INDIA	Indica		Arunachal Pradesh	*Pi54_13758*	0	4	0	0	3	2
22273	ARC 12707	INDIA	Javanica		Arunachal Pradesh	*Pi54_13758*	0	2	1	0	3	3
22300	ARC 12739	INDIA	Indica		Arunachal Pradesh	*Pi54_13758*	0	4	1	0	1	1
22315	ARC 12758	INDIA	Indica		Nagaland	*Pi54_13758*	1	5	3	3	-	0
22318	ARC 12761	INDIA	Javanica		Nagaland	*Pi54_13758*	0	3	1	0	5	0
22320	ARC 12764	INDIA	Japonica		Nagaland	*Pi54_13758*	0	6	2	1	3	2
22326	ARC 12770	INDIA	Javanica		Nagaland	*Pi54_22597*	0	5	4	1	1	3
22337	ARC 12785	INDIA	Javanica		Nagaland	*Pi54_22597*	0	4	0	1	8	1
22349	ARC 12803	INDIA	Javanica		Nagaland	*Pi54_22597*	0	1	0	0	1	1

22350	ARC 12804	INDIA	Indica		Nagaland	*Pi54_Tetep*	0	8	2	3	3	1
22363	ARC 12825	INDIA	Javanica		Nagaland	*Pi54_22597*	0	7	2	2	5	1
22368	ARC 12831	INDIA	Indica		Nagaland	*Pi54_Tetep*	0	6	1	2	3	0
22369	ARC 12832	INDIA	Indica		Nagaland	*Pi54_Tetep*	0	7	0	0	5	0
22375	ARC 12839	INDIA	Indica		Nagaland	*Pi54_Tetep*	1	6	1	1	5	0
22381	ARC 12845	INDIA	Javanica		Nagaland	*Pi54_13758*	0	6	0	0	-	0
22394	ARC 12860	INDIA	Javanica		Nagaland	*Pi54_22597*	0	6	5	0	2	8
22401	ARC 12867	INDIA	Indica		Nagaland	*Pi54_Tetep*	0	7	4	2	2	1
22409	ARC 12875	INDIA	Indica		Nagaland	*Pi54_Tetep*	0	5	3	0	2	2
22410	ARC 12876	INDIA	Javanica		Nagaland	*Pi54_22597*	0	7	1	1	6	1
22419	ARC 12886	INDIA	Javanica		Nagaland	*Pi54_22419*	1	4	1	0	5	0
22422	ARC 12889	INDIA	Javanica		Nagaland	*Pi54_22597*	0	5	1	1	7	1
22427	ARC 12895	INDIA	Javanica		Nagaland	*Pi54_22597*	0	1	3	0	6	1
22433	ARC 12903	INDIA	Indica		Nagaland	*Pi54_Tetep*	0	6	5	3	7	3
22446	ARC 12916	INDIA	Indica		Nagaland	*Pi54_22597*	0	2	-	4	1	8
22512	ARC 13154	INDIA	Japonica		Nagaland	*Pi54_13758*	0	2	3	0	8	1
22518	ARC 13160	INDIA	Indica		Nagaland	*Pi54_13758*	0	0	1	0	1	0
22522	ARC 13164	INDIA	Indica		Nagaland	*Pi54_13758*	0	5	2	1	2	0
22525	ARC 13167	INDIA	Javanica		Nagaland	*Pi54_13758*	0	5	1	1	2	2
22535	ARC 13177	INDIA	Indica		Nagaland	*Pi54_Tetep*	0	7	1	0	3	0
22540	ARC 13183	INDIA	Indica		Nagaland	*Pi54_10202*	0	1	3	1	2	1
22542	ARC 13185	INDIA	Javanica		Nagaland	*Pi54_22597*	0	1	2	1	1	1
22544	ARC 13187	INDIA	Javanica		Nagaland	*Pi54_22597*	0	1	3	1	1	0
22586	ARC 13234	INDIA	Javanica		Nagaland	*Pi54_22597*	0	8	4	0	3	1
22593	ARC 13241	INDIA	Javanica		Nagaland	*Pi54_22597*	0	5	4	0	5	1
22597	ARC 13245	INDIA	Indica		Nagaland	*Pi54_22597*	0	4	0	0	5	0
22600	ARC 13248	INDIA	Indica		Nagaland	*Pi54_40636*	0	5	0	1	5	1
22613	ARC 13262	INDIA	Javanica		Nagaland	*Pi54_22597*	0	2	0	1	3	0
22614	ARC 13263	INDIA	Indica		Nagaland	*Pi54_Tetep*	0	2	1	1	6	1
22622	ARC 13276	INDIA	Indica		Nagaland	*Pi54_22126*	0	6	3	2	3	3
22653	ARC 13321	INDIA	Indica		Nagaland	*Pi54_22126*	0	5	2	0	8	1
22679	ARC 13358	INDIA	Japonica		Nagaland	*Pi54_22126*	0	5	-	1	8	1
22698	ARC 13386	INDIA	Indica		Nagaland	*Pi54_22126*	0	5	2	1	1	0
22702	ARC 13393	INDIA	Indica		Nagaland	*Pi54_22597*	0	1	-	1	1	0
22705	ARC 13397	INDIA	Japonica		Nagaland	*Pi54_22597*	0	2	1	1	1	0
24243	CHAKLA 59	INDIA	Indica		NA	*Pi54_40996*	0	8	5	2	8	5
24256	ARC 10556	INDIA	Indica		Meghalaya	*Pi54_40996*	0	6	4	2	8	3
24258	ARC 10566	INDIA	Indica		Meghalaya	*Pi54_40996*	0	7	5	1	6	1
24266	ARC 11801	INDIA	Indica		Arunachal Pradesh	*Pi54_13758*	0	7	4	1	8	4
24270	ARC 12763	INDIA	Indica		Nagaland	*Pi54_22126*	0	5	2	0	3	1
32559	NP125	INDIA	Indica		NA	*Pi54_40636*	0	8	7	5	5	3
36764	VL 206	INDIA	Indica		NA	*Pi54_52694*	0	1	1	0	0	0
40636	KADA 176-12	INDIA	Indica		NA	*Pi54_40636*	0	8	6	3	8	3
40996	ARC 12034	INDIA	Indica		Arunachal Pradesh	*Pi54_40996*	0	5	0	7	2	5
41005	ARC 12135	INDIA	Indica		Arunachal Pradesh	*Pi54_40996*	0	5	1	1	3	2
41010	ARC 12141	INDIA	Indica		Arunachal Pradesh	*Pi54_40996*	0	1	0	1	5	1
41014	ARC 12167	INDIA	Indica		Arunachal Pradesh	*Pi54_40996*	0	1	0	0	2	0
41015	ARC 12175	INDIA	Indica		Arunachal Pradesh	*Pi54_40996*	0	5	0	1	3	1
41044	ARC 12362	INDIA	Indica		Arunachal Pradesh	*Pi54_22126*	0	2	3	1	6	3
41075	ARC 12592	INDIA	Indica		Arunachal Pradesh	*Pi54_40996*	0	7	3	4	7	3
41084	ARC 12671	INDIA	Indica		Arunachal Pradesh	*Pi54_40996*	0	5	0	7	3	3
41112	ARC 13380	INDIA	Indica		Nagaland	*Pi54_22126*	0	7	2	5	6	1
41117	ARC 13421	INDIA	Indica		Arunachal Pradesh	*Pi54_40996*	1	3	1	1	3	1

41142	ARC 13526	INDIA	Indica		Arunachal Pradesh	*Pi54_40996*	1	7	0	8	7	5
41150	ARC 13535	INDIA	Indica		Arunachal Pradesh	*Pi54_40996*	0	6	4	7	3	3
41195	ARC 13663	INDIA	Indica		Arunachal Pradesh	*Pi54_40996*	0	6	1	3	5	1
41217	ARC 13782	INDIA	Indica		Assam	*Pi54_40996*	0	6	4	2	5	1
41233	ARC 13814	INDIA	Indica		Assam	*Pi54_40996*	0	7	1	3	7	1
41283	ARC 13884	INDIA	Indica		Assam	*Pi54_40996*	1	4	3	3	7	1
41285	ARC 13886	INDIA	Indica		Assam	*Pi54_40996*	0	4	3	3	6	1
41294	ARC 13897	INDIA	Indica		Assam	*Pi54_40996*	0	7	2	2	7	0
41297	ARC 13900	INDIA	Indica		Assam	*Pi54_40996*	1	3	1	2	5	1
41299	ARC 13902	INDIA	Indica		Assam	*Pi54_40996*	0	6	3	1	8	1
41305	ARC 13909	INDIA	Indica		Assam	*Pi54_40996*	0	1	1	0	5	0
41307	ARC 13911	INDIA	Indica		Assam	*Pi54_40996*	0	6	1	1	8	1
41308	ARC 13912	INDIA	Indica		Assam	*Pi54_40996*	0	1	2	3	7	2
41311	ARC 13915	INDIA	Indica		Assam	*Pi54_40996*	0	5	3	5	7	1
41325	ARC 13934	INDIA	Indica		Assam	*Pi54_40996*	0	7	2	5	7	1
41326	ARC 13935	INDIA	Indica		Assam	*Pi54_40996*	0	9	3	5	8	1
41327	ARC 13938	INDIA	Indica		Assam	*Pi54_40996*	0	6	3	7	7	1
41328	ARC 13939	INDIA	Indica		Assam	*Pi54_40996*	0	5	3	1	8	2
41356	ARC 14037	INDIA	Indica		Assam	*Pi54_40996*	0	1	2	0	1	6
41364	ARC 14047	INDIA	Indica		Assam	*Pi54_40996*	0	5	5	1	2	7
41368	ARC 14052	INDIA	Indica		Assam	*Pi54_40996*	1	3	2	1	3	7
41375	ARC 14061	INDIA	Indica		Assam	*Pi54_40996*	0	7	3	5	1	7
41395	ARC 14094	INDIA	Indica		Assam	*Pi54_40996*	0	6	1	2	0	4
41508	ARC 14302	INDIA	Indica		Nagaland	*Pi54_40996*	1	7	5	2	8	7
41509	ARC 14320	INDIA	Indica		Assam	*Pi54_40636*	0	1	1	0	0	4
41535	ARC 14382	INDIA	Indica		Assam	*Pi54_40996*	0	5	4	1	8	6
41539	ARC 14389	INDIA	Indica		Assam	*Pi54_40996*	0	5	1	3	8	7
41582	ARC 14520	INDIA	Indica		Assam	*Pi54_40996*	0	7	1	4	8	7
41615	ARC 14577	INDIA	Indica		Assam	*Pi54_40996*	0	7	0	3	6	1
41616	ARC 14578	INDIA	Indica		Assam	*Pi54_40996*	0	8	2	4	6	3
41617	ARC 14579	INDIA	Indica		Assam	*Pi54_40996*	0	7	4	3	7	5
41620	ARC 14582	INDIA	Indica		Assam	*Pi54_40996*	0	7	3	3	8	5
41625	ARC 14594	INDIA	Indica		Assam	*Pi54_40996*	0	5	5	2	6	7
41628	ARC 14603	INDIA	Indica		Assam	*Pi54_40996*	0	5	7	1	7	6
41633	ARC 14609	INDIA	Indica		Assam	*Pi54_40996*	1	7	2	1	5	1
41655	ARC 14642	INDIA	Indica		Assam	*Pi54_40996*	0	1	0	0	0	1
41690	ARC 14690	INDIA	Indica		Assam	*Pi54_40996*	0	8	2	5	7	7
41699	ARC 14702	INDIA	Indica		Assam	*Pi54_40996*	0	5	2	7	4	6
41732	ARC 14740	INDIA	Indica		Tripura	*Pi54_40996*	0	7	1	5	4	0
41736	ARC 14756	INDIA	Indica		Tripura	*Pi54_40636*	0	1	6	1	2	0
41744	ARC 14770	INDIA	Indica		Tripura	*Pi54_22597*	0	3	2	4	5	1
41818	ARC 14915	INDIA	Indica		Tripura	*Pi54_41818*	0	8	3	1	7	5
41831	ARC 14948	INDIA	Indica		Tripura	*Pi54_22126*	0	6	9	2	7	5
41844	ARC 14969	INDIA	Indica		Tripura	*Pi54_Tetep*	0	6	8	2	8	2
41882	ARC 15033	INDIA	Indica		Manipur	*Pi54_40636*	0	6	6	1	7	7
41895	ARC 15046	INDIA	Indica		Manipur	*Pi54_22126*	0	0	0	0	1	2
41938	ARC 15129	INDIA	Indica		Manipur	*Pi54_40636*	0	5	3	1	2	3
41964	ARC 15222	INDIA	Indica		Manipur	*Pi54_40996*	0	7	0	4	2	2
42014	ARC 15335	INDIA	Indica		Manipur	*Pi54_13758*	0	3	1	1	1	1
42116	ARC 15691	INDIA	Indica		Manipur	*Pi54_40996*	0	8	1	4	6	3

42158	ARC 15890	INDIA	Indica		Assam	*Pi54_40996*	0	3	0	3	2	0
42165	ARC 15905	INDIA	Indica		Assam	*Pi54_40996*	0	8	3	5	7	6
42172	ARC 15912	INDIA	Indica		Assam	*Pi54_40996*	0	2	1	0	6	6
42173	ARC 15913	INDIA	Indica		Assam	*Pi54_40996*	0	2	1	0	6	4
42220	ARC 18039	INDIA	Indica		NA	*Pi54_40996*	1	7	5	1	8	3
42221	ARC 18040	INDIA	Indica		NA	*Pi54_40996*	0	6	2	2	6	1
42245	ARC 18075	INDIA	Indica		NA	*Pi54_40996*	0	3	1	1	1	1
42263	ARC 18101	INDIA	Indica		NA	*Pi54_40996*	0	5	1	0	-	2
42264	ARC 18102	INDIA	Indica		NA	*Pi54_40996*	0	6	0	0	3	3
42293	ARC 18134	INDIA	Indica		NA	*Pi54_40996*	0	6	6	1	8	6
42307	ARC 18152	INDIA	Indica		NA	*Pi54_40996*	0	5	2	1	5	5
42308	ARC 18153	INDIA	Indica		NA	*Pi54_40996*	0	5	2	1	6	6
42315	ARC 18173	INDIA	Indica		NA	*Pi54_40996*	0	7	0	1	6	6
42319	ARC 18182	INDIA	Indica		NA	*Pi54_40996*	0	8	8	3	8	8
42320	ARC 18184	INDIA	Indica		NA	*Pi54_22126*	0	7	2	1	6	4
42321	ARC 18191	INDIA	Indica		NA	*Pi54_40996*	0	7	7	2	7	8
42326	ARC 18198	INDIA	Indica		NA	*Pi54_40996*	0	9	2	0	8	4
42335	ARC 18217	INDIA	Indica		NA	*Pi54_22126*	0	6	5	1	5	4
42336	ARC 18218	INDIA	Indica		NA	*Pi54_22126*	0	7	3	2	3	4
42339	ARC 18223	INDIA	Indica		NA	*Pi54_40996*	0	6	5	3	3	5
42340	ARC 18224	INDIA	Indica		NA	*Pi54_40996*	0	6	3	1	2	2
42383	ARC 18303	INDIA	Indica		NA	*Pi54_40996*	0	3	2	3	2	1
42385	ARC 18307	INDIA	Indica		NA	*Pi54_40996*	0	7	3	2	2	6
42412	ARC 18347	INDIA	Indica		NA	*Pi54_Tetep*	0	6	4	3	5	7
42423	ARC 18371	INDIA	Indica		NA	*Pi54_40996*	0	6	5	3	7	7
42425	ARC 18375	INDIA	Indica		NA	*Pi54_40996*	1	7	1	2	6	6
42439	ARC 18517	INDIA	Indica		NA	*Pi54_42439*	0	8	2	2	8	6
42482	ARC 5933	INDIA	Indica		Assam	*Pi54_40996*	0	7	6	3	8	7
42601	ARC 10852	INDIA	Indica		Meghalaya	*Pi54_40996*	0	8	1	0	6	1
42664	ARC 11397	INDIA	Indica		Arunachal Pradesh	*Pi54_13758*	0	6	1	0	7	3
42676	ARC 11551	INDIA	Indica		Arunachal Pradesh	*Pi54_40996*	0	7	6	0	8	7
42677	ARC 11553	INDIA	Indica		Arunachal Pradesh	*Pi54_40996*	0	8	7	0	8	7
42678	ARC 11557	INDIA	Indica		Arunachal Pradesh	*Pi54_40996*	0	6	1	0	6	4
42679	ARC 11565	INDIA	Indica		Arunachal Pradesh	*Pi54_40996*	0	6	5	1	8	6
42703	ARC 11682	INDIA	Indica		Arunachal Pradesh	*Pi54_40996*	0	3	2	7	5	5
42734	ARC 13510	INDIA	Indica		Arunachal Pradesh	*Pi54_40996*	0	2	0	1	5	6
42770	ARC 13760	INDIA	Indica		assam	*Pi54_22126*	0	-	0	0	-	-
42792	ARC 13800	INDIA	Indica		assam	*Pi54_40996*	0	7	3	4	6	3
42821	ARC 13982	INDIA	Indica		assam	*Pi54_40996*	0	6	6	0	8	3
42832	ARC 13998	INDIA	Javanica		assam	*Pi54_40996*	0	1	0	0	0	0
42833	ARC 14000	INDIA	Indica		assam	*Pi54_40996*	0	5	5	2	2	3
42836	ARC 14004	INDIA	Indica		assam	*Pi54_40996*	0	4	2	5	5	7
42838	ARC 14008	INDIA	Indica		assam	*Pi54_40996*	0	5	1	4	4	7
42844	ARC 14023	INDIA	Indica		assam	*Pi54_40996*	0	7	2	5	6	1
42921	ARC 14422	INDIA	Indica		assam	*Pi54_40996*	0	8	3	4	4	6
42922	ARC 14423(MEDIUM GRAIN)	INDIA	Indica		assam	*Pi54_22597*	0	-	-	0	6	1
43035	ARC 14967	INDIA	Indica		Tripura	*Pi54_22126*	0	5	1	4	6	3
43211	ARC 15724	INDIA	Indica		Manipur	*Pi54_40996*	0	7	0	0	3	1
45037	BAKTULSI	INDIA	Indica		NA	*Pi54_41818*	0	5	3	1	8	5
45043	BALAM	INDIA	Indica		NA	*Pi54_41818*	0	8	4	2	8	7

45093	BASANTOBAHAR	INDIA	Indica		NA	*Pi54_22126*	0	8	6	3	8	7
45115	BEGUNBICHI	INDIA	Indica		NA	*Pi54_22126*	0	7	2	4	8	6
45151	BHASAMANIK(LOCAL)	INDIA	Indica		NA	*Pi54_40636*	0	6	1	6	5	4
45212	BONA	INDIA	Indica		NA	*Pi54_22126*	0	0	0	3	1	4
45226	BORO AUS 1585-35	INDIA	Indica		NA	*Pi54_22597*	0	5	2	1	5	1
45272	CHANDANSAIL	INDIA	Indica		NA	*Pi54_10202*	0	5	1	1	3	5
45284	CHENGA	INDIA	Indica		NA	*Pi54_40636*	0	7	5	2	7	7
45305	CHINSURAH	INDIA	Indica		NA	*Pi54_40636*	0	3	3	2	4	2
45310	CHOTASONI	INDIA	Indica		NA	*Pi54_Tetep*	0	7	4	1	7	1
45500	DEOLA	INDIA	Indica		NA	*Pi54_Tetep*	0	6	3	4	8	6
45530	DHAPSAIL	INDIA	Indica		NA	*Pi54_Tetep*	0	8	4	3	4	4
45807	HORKASAIL	INDIA	Indica		NA	*Pi54_52694*	0	5	7	1	8	7
45852	JAGAL	INDIA	Indica		NA	*Pi54_Tetep*	0	7	7	1	8	8
45946	KABILRASH	INDIA	Indica		NA	*Pi54_41818*	0	4	6	3	8	6
46028	KANAKCHUR	INDIA	Indica		NA	*Pi54_10202*	0	0	0	8	2	1
46066	KASHIFUL	INDIA	Indica		NA	*Pi54_40636*	0	1	0	0	0	0
46102	KELE	INDIA	Indica		NA	*Pi54_Tetep*	0	8	7	3	7	7
46123	KHASH	INDIA	Indica		NA	*Pi54_10202*	0	5	1	1	5	5
46632	SAKET 1	INDIA	Indica		NA	*Pi54_Tetep*	0	6	2	1	8	4
46837		INDIA	Indica		NA	*Pi54_Tetep*	0	5	2	3	2	1
46879		INDIA	Indica		NA	*Pi54_10202*	0	6	0	5	4	1
47580	BROWN GORA S.N. 27	INDIA	Indica		NA	*Pi54_22126*	0	7	3	3	7	3
49558	CUL 14633	INDIA	Indica		Tamil Nadu	*Pi54_40636*	0	5	1	5	6	4
49560	CUTTACK 3	INDIA	Indica		Odisha	*Pi54_22126*	0	7	6	1	8	7
49561	CUTTACK 5	INDIA	Indica		Odisha	*Pi54_22126*	0	8	6	1	8	7
49729	JOHARI	INDIA	Indica		Assam	*Pi54_40996*	0	7	1	2	6	6
49733	KALA OR BLACK AMON(DW)	INDIA	Indica		NA	*Pi54_40636*	2	7	1	3	6	7
49743	KAL MANAVARI 2(TINNY 6-1)	INDIA	Indica		Tamil Nadu	*Pi54_22597*	0	8	2	1	7	7
49778	KASTURI KAZAMA	INDIA	Indica		Tamil Nadu	*Pi54_22126*	0	1	2	2	8	4
49850	LAWANGAI	INDIA	Indica		NA	*Pi54_40636*	0	5	5	1	7	6
49856	LOCAL PADDY	INDIA	Indica		NA	*Pi54_40996*	0	7	3	3	6	4
52694	MADHUKAR	INDIA	Indica	Landrace/Traditional cultivar	NA	*Pi54_52694*	0	3	2	1	7	1

NA: Not available; UBN-Uniform Blast Nursery; *information from IRGCIS.

Scoring was based on 0-9 scale of standard evaluation system for leaf blast used in IRRI, Philippines. Score 0-3 considered as blast resistant.
